# Ocular Manifestations of Takayasu’s Arteritis—A Case-Based Systematic Review and Meta-Analysis

**DOI:** 10.3390/jcm12113745

**Published:** 2023-05-29

**Authors:** Urszula Szydełko-Paśko, Joanna Przeździecka-Dołyk, Łukasz Nowak, Artur Małyszczak, Marta Misiuk-Hojło

**Affiliations:** 1 Department of Ophthalmology, Wrocław Medical University, 50-556 Wrocław, Poland; 2 Department of Optics and Photonics, Wrocław Univeristy of Science and Technology, 50-370 Wrocław, Poland; 3 Department of Minimally Invasive and Robotic Urology, University Center of Excellence in Urology, Wrocław Medical University, 50-367 Wrocław, Poland

**Keywords:** Takayasu’s arteritis, pulseless disease, ocular manifestations, retinal ischemia, retinal artery occlusion, retinal vein occlusion, uveitis, optic neuropathy

## Abstract

Takayasu’s arteritis (TA) is a type of vasculitis in which inflammation develops in large vessels, especially in the aorta and its branches. Our study aims to determine the prevalence and type of ocular manifestations in TA. A systematic literature search was conducted in December 2022 using three electronic databases (PubMed, Scopus, and Web of Science). The following data were extracted from each article: the name of the first author; the patient’s age, sex, and origin (continent); circumstances connected with the diagnosis of TA; symptoms given by the patients; reported ocular manifestations; and administered treatment. The final analysis was based on data collected from 122 cases. Retinal ischemia, followed by optic neuropathy, cataract, and retinal artery occlusion, were the most prevalent eye conditions associated with the disease. Systemic steroid therapy, vascular procedures, and methotrexate were mainly used to treat pulseless disease. Patients mostly complained of gradual vision acuity loss, sudden vision acuity loss, ocular pain, and amaurosis fugax. The diagnosis of Takayasu’s arteritis should be considered in patients presenting symptoms of visual decline/loss, ocular pain, or signs of retinal ischemia, optic neuropathy, or early cataract formation. A proper diagnosis is crucial to ensure the patient receives treatment without significant delay.

## 1. Introduction

Numerous systematic reviews have been devoted to ocular manifestations of various systemic diseases. Not many of them, however, have been concerned with the involvement of the eye in Takayasu’s arteritis (TA). In 2021, Turk et al. published a systematic review and meta-analysis concerning ocular findings in diseases such as rheumatoid arthritis, connective tissue diseases, and vasculitis, including giant cell arteritis and granulomatosis polyangiitis [1]. Subsequently, in another systematic review and meta-analysis, Turk et al. described the ocular manifestations of Behcet’s disease [2]. Even though the association between the eye and TA has been pointed out in some previous review articles, a systematic review summarising the current evidence has not been published to date. Therefore, we sought to perform a systematic review and meta-analysis to determine the prevalence and type of ocular manifestations in TA.

TA is a type of vasculitis in which inflammation develops in large vessels, especially in the aorta and its branches [3]. A chronic inflammatory process eventually leads to the obliteration and narrowing of the blood vessels [4]. The prevalence of the disease varies from 0.9 to 40 cases per million; however, according to epidemiological data, the number is estimated to be between 4 and 15 cases per million [5]. Geographical and ethnic differences might explain this wide range. TA is more common in Asian, Central, and South American populations. In addition, women are more frequently affected by the disease, with the women-to-men ratio being 1.6–12:1 in adults [5]. The highest incidence occurs in the third decade, although the disease may develop even in infancy [5]. The pathogenesis of TA is not fully known; however, autoimmune processes and genetic factors are believed to be significant [6,7].

## 2. Methods

### 2.1. Search Strategy

The present study was performed according to the Preferred Reporting Items for Systematic Reviews and Meta-Analysis (PRISMA) statement [8]. A systematic literature search was conducted independently by two authors (USP and JPD) using three electronic databases (PubMed, Scopus, and Web of Science). Any disagreements were discussed with a third author (MMH). The combination of the following Medical Subject Headings (MeSH), keywords, and phrases was used: (“Takayasu’s arteritis” OR “pulseless disease” OR “aortoarteritis”) AND (“ocular” OR “ophthalmology” OR “eye” OR “ocular manifestations”). No language and publication time restrictions were applied. The last search was conducted on 15 December 2022. The references of the included articles were also examined in order to expand the eligible sources.

### 2.2. Inclusion and Exclusion Criteria

In the present study, we included studies reporting data from patients with previously or newly diagnosed TA who presented any ocular manifestation. Data from case reports, case series, clinical images and letters to the editors were found to be eligible. The coexistence of TA with any other vasculitis or autoimmune disease was the first exclusion criterion. Another exclusion criterion was no description of an ophthalmological examination. Studies in which only subjective visual disturbances were reported by the patients, as well as reports of ocular manifestations secondary to the administered treatment, were additionally excluded. Reviews, conference abstracts and animal studies were also rejected, as well as studies on a large number of patients as they were sparse and incomplete.

### 2.3. Data Extraction

The following data were extracted from each article: the name of the first author, patient’s age, sex, and origin (continent), circumstances connected with the diagnosis of TA, symptoms given by the patients, reported ocular manifestation, and administered treatment. The disease diagnosis was either new for the patient or made before the diagnostic period described in a specific article. Only the systemic treatment for TA was taken into account. Specific treatments connected with various ocular manifestations of the disease were not presented.

### 2.4. Statistical Analysis

Data were analysed using Statistica 13.3 software (Tibco Software Inc., Palo Alto, CA, USA). We gathered individual patient data from each study. Categorical variables (patient characteristics, ocular manifestations, treatment and symptoms) were summarized descriptively and presented as counts and percentages. A subset of analyses was performed to determine the potential differences in the occurrence of ocular manifestations of Takayasu’s arteritis concerning gender (male vs. female) and age (children vs. adults). In a subgroup analysis, categorical variables were assessed by the Chi-squared test or Fisher’s exact test. A *p*-value ≤ 0.05 was considered statistically significant.

## 3. Results

**Figure 1:**
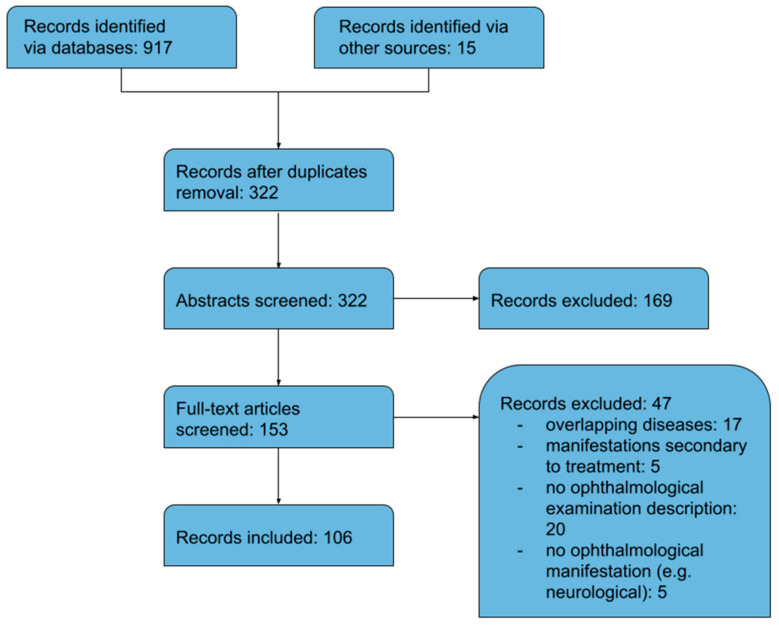
Flow diagram of meta-analysis.

The detailed flow diagram of the study selection process (with subsequent exclusions) is presented in Figure 1. The literature search identified 932 references. All citations were exported to the citation manager EndNote 20 (Clarivate Analytics), and duplicate references (*n* = 322) were removed. After screening the titles and abstracts, 169 studies were excluded due to their irrelevance to the current topic. The full text of 153 articles was read in detail to determine their eligibility. Subsequently, 47 articles were excluded for the following reasons: overlapping diseases (17), ocular manifestations secondary to treatment (5), no ophthalmological examination (20), and no ophthalmological manifestations (5). Eventually, 106 records were included in the analysis. The included records consisted of five case series, seven letters to editors, one photo essay, one clinical image, and 92 case reports. Selected articles provided data on 122 patients, as summarised in Table 1, Table 2, Table 3 and Table 4 (Supplementary Tables S1–S3). The age distribution of patients with ocular manifestations of TA is presented in Table 5.

The mean age of patients with ocular manifestations of Takayasu’s arteritis was 31.4 years, with a female-to-male ratio of 4.8:1. The vast majority were aged between 11 and 40 (76.3%) with the peak in the third decade (33.6%). Most patients were from Asia (23.8%). In over 74% of cases, the ocular manifestations preceded the diagnosis of TA. The most common eye disorder accompanying the disease was retinal ischemia (present in 57.4% of patients), followed by optic neuropathy (18%), cataract (14.8%), and retinal artery occlusion (12.3%). Systemic steroid therapy, vascular procedures, and methotrexate were predominantly used as primary treatments for TA (68.9%, 32%, and 27%, respectively). Patients mostly complained of gradual vision acuity loss (52.5%), sudden vision acuity loss (23%), ocular pain (17.2%), and amaurosis fugax (25.4%), with the last one described as transient blurring, fogging, dimming, seeing shades/curtains or “white out”. Children (under the age of 18) presented mainly with retinal ischemia (57.1%) and uveitis (28.5%). Out of five cases of uveitis reported in the course of TA, four concerned children. The difference in the prevalence of uveitis in children and adults was statistically significant (*p* < 0.05). The occurrence of other ocular manifestations and symptoms of TA was similar in children and adults. No statistically significant difference in the prevalence of any ocular manifestation of TA was found in the gender subgroup analysis (men vs. women).

## 4. Discussion

The first description of a patient suffering from TA dates back to 1908 and is attributed to Mikito Takayasu [114]. The Japanese ophthalmologist had observed malformations of the retinal blood vessels and shared his findings with fellow physicians, who ascribed similar findings to patients with an impalpable pulse. A classification of specific retinal disorders was introduced in 1976 by Uyama and Asayama [115]. In 1990, the American College of Rheumatology published criteria for the classification of TA to facilitate the diagnostic process [116]. However, none of the criteria refers to changes in the eye. So even though the eyes of patients with pulseless disease are known to be affected by the systemic condition, no ophthalmological examination is required when establishing a diagnosis. Therefore, some severe ocular manifestations may go unnoticed for an extended time.

Based on the results of our study, the characteristics of patients with ocular manifestations were consistent with the profile of a patient with TA without any eye disorders. Most cases described young women diagnosed and treated in Asia. The majority of patients were in the third decade of their lives, followed by those in their 30 s and teenagers. Although the age range between 20 and 40 is typical for the disease, TA may also affect children. The youngest reported case was 5 years old. Ocular disorders present in relatively young patients should be particularly concerning, as they usually cannot be attributed to other comorbidities that are commonly associated with advanced age.

What seems particularly interesting is that in 91 cases (74.6%), the diagnosis of TA was made after the patients had noticed some ocular problems. The first symptoms of TA might be non-specific (e.g., malaise, fever, fainting) and attributed to many conditions. For this reason, the diagnosis of the disease is challenging and may leave the patient unalarmed for a long time. However, visual decline/loss, ocular pain, or redness of the eyes can prompt the patient to seek professional medical consultation. That is why ophthalmologists should be aware of the ocular manifestations of pulseless disease, especially in relatively young people without an obvious underlying cause. They should run detailed diagnostics and reach out to angiologists or rheumatologists to ensure comprehensive medical care.

Most patients presented with features of retinal ischemia caused by insufficient blood supply due to the narrowing and fibrosis of the aorta and its branches. In 15 cases, the ocular ischemic syndrome was described as a result of carotid artery stenosis. Even though hypoperfusion retinopathy seems typical of the disease, one must not forget that hypertensive retinopathy may also develop as a result of renal artery stenosis. Therefore, while analysing the results of imaging studies, it may be possible to predict what type of ocular disorder a patient can develop based on the branch of the aorta affected by the disease.

The changes in the blood flow in the course of the disease are secondary to an inflammatory process. Our research has shown that some patients with TA developed ocular manifestations such as scleritis, episcleritis, uveitis, keratitis, which are typically associated with autoimmune diseases. A systemic inflammatory response may lead to a local manifestation of the disease. Bolek et al. reported that in children with TA, inflammation was more severe than in adults [117]. This may partially explain why four out of five cases of uveitis concerned children.

The diagnosis of TA in children is challenging indeed. In Clemente et al.’s material, 26.8% of pediatric TA cases were initially misdiagnosed [118]. As time is of the essence, the sooner the accurate diagnosis is made and the appropriate treatment administered, the smaller the chances of irreversible damage. Ueno et al. described a complete resolution of retinal ischemic features in the fundus in a 17-year-old girl thanks to a proper diagnosis and successful vascular procedure [110].

In most cases analysed in our study (68.9%), systemic steroids were used to induce remission. Additionally, some patients were prescribed immunosuppressants (methotrexate, azathioprine, mycophenolate mofetil, cyclophosphamide, and leflunomide). This combination is consistent with the 2018 EULAR recommendations for the management of large vessel vasculitis [119]. Zeng et al. also proposed a similar algorithm to cope with ocular ischemia in the course of TA [113]. Moreover, their systematic review suggested surgical intervention in conjunction with medications in order to achieve the best results. According to our results, vascular procedures were performed in only 32% of cases. However, it should be noted that some cases date back to the second half of the previous century, and not all of the operative techniques were commonly used then. Furthermore, several patients refused to undergo surgery.

The main limitation of this paper is the inclusion of only case reports and case series. However, we are mainly interested in diseases of low prevalence, for which there are only a few incomplete original studies on a large number of patients (“incomplete” in the sense that some crucial information is missing such as patients’ comorbidities, the diagnostics and administered treatment). Clinical knowledge of rare diseases often relies on case reports, which can have significant implications for healthcare decision-making. Conducting future multicenter research involving a large number of patients could provide clinicians with guidelines on managing ocular manifestations of TA.

## 5. Conclusions

Ophthalmologists must be aware of the possible ocular symptoms of TA to ensure the patient gets a correct diagnosis and treatment without significant delay. Young people presenting with ocular disorders and without any known comorbidities should be particularly alert. The multidisciplinary cooperation between ophthalmologists, angiologists, and rheumatologists is crucial, as eye disorders may be the first sign of TA. Raising awareness among patients with TA about possible ocular complications is also crucial to accelerating the administration of appropriate treatment. Conducting screenings for patients with pulseless disease may be beneficial in detecting early abnormalities.

## Figures and Tables

**Table 1:** Characteristics of patients with TA’s ocular manifestations.

First Author	Age	Sex	Patient’s Origin (Continent)	New Diagnosis of TA
Akhtar [9]	35	M	-	No
Amer [10]	26	M	-	No
Anguita [11]	12	F	South America	Yes
Arya [12]	45	F	-	No
Austen [13]	28	F	Asia	Yes
Babu [14]	31	F	-	Yes
Bajgai [15]	25	F	-	Yes
Balaskas [16]	35	F	Europe	No
Bapat [17]	24	M	-	Yes
Batliwala [18]	18	F	Europe	No
Becker [19]	5	F	-	Yes
Bodker [20]	22	F	South America	Yes
Bouzas [21]	45	F	Europe	No
Caccamise [22]	19	F	-	Yes
Chaudhary [23]	44	F	South America	Yes
Chawla [24]	28	M	Asia	Yes
Christiansen [25]	19	F	Asia	Yes
Conrath [26]	28	F	Africa	Yes
Das [27]	37	F	-	Yes
Demir [28]	14	F	-	Yes
Do Vale [29]	46	F	-	Yes
Dowling [30]	54	-	-	Yes
Elizalde [31]	63	F	-	No
Escano [32]	34	M	-	No
Font [33]	35	F	Asia	Yes
Gaur [34]	27	F	-	Yes
Genc [35]	52	M	-	Yes
Gong [36]	18	F	Asia	Yes
Guclu [37]	48	F	-	Yes
Gupta [38]	18	M	-	Yes
Harada [39]	34	F	Asia	Yes
Hayasaka [40]	33	F	Asia	No
Hayasaka [40]	19	F	Asia	Yes
Herath [41]	38	F	Asia	Yes
Ibrahim [42]	39	F	-	No
Jain [43]	40	F	Asia	Yes
Jain [44]	39	F	Asia	Yes
Jain [45]	15	F	-	No
Kaliaperumal [46]	35	F	-	Yes
Kancherla [47]	27	F	-	Yes
Kannan [48]	13	M	Asia	Yes
Kapran [49]	29	M	-	Yes
Karam [50]	28	F	-	Yes
Karam [50]	24	F	-	Yes
Karam [50]	27	F	-	Yes
Karam [50]	24	F	-	Yes
Karam [50]	21	F	-	Yes
Karam [50]	8	F	-	Yes
Karam [50]	30	F	-	Yes
Karwatowski [51]	66	F	Asia	No
Kaushik [52]	40	F	-	Yes
Kausman [53]	12	M	Asia	Yes
Kavitha [54]	40	F	-	Yes
Kim [55]	25	F	Asia	Yes
Kimura [56]	41	M	-	No
Kinoshita [57]	28	F	-	No
Koz [58]	45	M	-	Yes
Kumar [59]	65	M	-	Yes
Kuwahara [60]	57	F	Asia	No
Larrazabal [61]	26	F	-	Yes
Lee [62]	23	F	-	Yes
Lee [63]	28	F	Asia	Yes
Leonard [64]	37	F	-	Yes
Lewis [65]	59	F	-	Yes
Lim [66]	53	F	-	No
Mahajan [67]	36	F	-	Yes
Mahajan [67]	14	F	-	Yes
Mahajan [67]	34	F	-	Yes
Mahajan [67]	25	F	-	Yes
Mahajan [67]	30	F	-	Yes
Mahendradas [68]	18	F	-	No
Mashru [69]	31	F	-	Yes
Matalia [70]	16	F	-	Yes
Matsumoto-Otake [71]	31	F	-	Yes
McDonald [72]	12	M	Asia	Yes
Milea [73]	32	F	Africa	No
Moncada [74]	32	F	Asia	Yes
Nithyanandam [75]	30	F	-	No
Noel [76]	58	F	-	Yes
Noel [76]	48	M	-	No
Noel [76]	58	F	-	No
Ostler [77]	47	M	-	Yes
Padhy [78]	35	M	-	No
Pahwa [79]	27	F	Asia	No
Pallangyo [80]	24	F	Africa	Yes
Paterson [81]	25	F	-	Yes
Paul [82]	30	F	Asia	No
Pelegrin [83]	42	M	Asia	Yes
Peter [84]	37	F	-	Yes
Peter [85]	25	F	-	No
Peter [85]	29	F	-	Yes
Peter [85]	13	F	-	Yes
Peter [85]	28	M	-	No
Rainer [86]	30	F	-	Yes
Rahman [87]	22	F	Asia	No
Rajshri [88]	50’s	M	-	Yes
Ramteke [89]	48	F	-	Yes
Reddy [90]	27	F	-	No
Rodriguez [91]	26	F	-	Yes
Sakthiswary [92]	20	F	-	Yes
Santhanam [93]	19	F	-	Yes
Setty [94]	23	F	-	Yes
Setty [94]	23	F	-	Yes
Shailaja [95]	22	F	-	Yes
Shrestha [96]	20	F	-	Yes
Shukla [97]	44	F	Asia	Yes
Smith [98]	36	F	Asia	Yes
Stone [99]	13	F	North America	Yes
Strauss [100]	25	F	Asia	Yes
Subira [101]	32	F	-	Yes
Suematsu [102]	34	F	-	Yes
Suh [103]	52	F	-	Yes
Sureja [104]	48	F	-	Yes
Tani [105]	42	M	-	Yes
Taylan [106]	40	F	-	Yes
Tian [107]	23	F	Asia	Yes
Topcuoglu [108]	42	F	-	Yes
Torun [109]	20	F	-	No
Ueno [110]	17	F	Asia	No
Wu [111]	13	F	-	Yes
Zahaf [112]	20	F	-	Yes
Zeng [113]	29	F	Asia	No

**Table 2:** Ophthalmological symptoms reported by patients with TA.

Ophthalmological Symptoms	Number of Cases	% of Cases
Amaurosis fugax	31	25.4
Diplopia	2	1.6
Orbital pain	4	3.3
Ocular pain	21	17.2
Gradual visual acuity decrease	64	52.5
Sudden visual acuity decrease/loss	28	23.0
Ocular redness	16	13.1
Photophobia	10	8.2
Metamophopsia	1	0.8
Xanthopsia	1	0.8
No eye movement	1	0.8

**Table 3:** Ocular manifestations of TA.

Ocular Manifestation	Number of Cases	% of Cases
Retinal ischemia	70	57.4
Hypertensive retinopathy	7	5.7
Scleritis	9	7.4
Keratitis	3	2.5
Atypical Tolosa Hunt syndrome	1	0.8
Retinal artery occlusion	15	12.3
Cataract	18	14.8
Uveitis	5	4.1
Epiretinal membrane	1	0.8
Episcleritis	1	0.8
Optic neuropathy	22	18.0
Facial nerve palsy	2	1.6
Acquired ocular motor apraxia	1	0.8
Retinal vein occlusion	4	3.3
Ptosis	2	1.6
Ophthalmoplegia	1	0.8
Retinal vasculitis	5	4.1
Sterile corneal melt	1	0.8
Multiple evanescent white dot syndrome	1	0.8
Orbital pseudotumor	1	0.8
Exudative retinal detachment	1	0.8

**Table 4:** Systemic treatment used in patients with ocular manifestations of TA.

Systemic Treatment	Number of Cases	% of Cases
Systemic steroids	84	68.9
Vascular procedure	39	32.0
Mycophenolate mofetil	6	4.9
Azathioprine	8	6.6
Cyclophosphamide	7	5.7
Methotrexate	33	27.0
Leflunomide	1	0.8
Acetylsalicylic acid	22	18.0
Monoclonal antibody	9	7.4
Antiplatelet drugs	9	7.4
Anticoagulants	9	7.4
Antihypertensives	12	9.8

**Table 5:** Age distribution of patients with TA’s ocular manifestations.

Age Range	Number of Patients	% of Patients
0–10	2	1.6
11–20	24	19.7
21–30	41	33.6
31–40	28	23.0
41–50	15	12.3
51–60	9	7.4
61–70	3	2.5

## Data Availability

Data sharing is not applicable.

## Supplementary Materials

The following supplementary material can be downloaded at https://www.mdpi.com/article/10.3390/jcm12113745/s1, Table S1. Ophthalmological symptoms reported by TA patients. Table S2. Ocular manifestations of TA. Table S3. Systemic treatment in patients with ocular manifestations of TA.
