# Supplementary material for: Ocular Manifestations of Takayasu’s Arteritis—A Case-Based Systematic Review and Meta-Analysis
Source: J Clin Med. 2023 May 29;12(11):3745. doi: 10.3390/jcm12113745 (PMC10253919; doi:10.3390/jcm12113745)
Supplement: Supplementary file 1 [file jcm-12-03745-s001.zip › jcm-2326701-supplementary.pdf]

Table S1. Ophthalmological symptoms reported by TA patients.

First author	Amaurosis fugax	Diplopia	Orbital pain	Ocular pain	Gradual VA decrease	Sudden VA decrease/loss	Ocular redness	Photophobia	Metamorphopsia	Xanthopsia	No eye movement
Akhtar	0	0	0	1	0	0	0	0	0	0	0
Amer	0	0	0	1	1	0	1	1	0	0	0
Anguita	1	0	0	0	0	0	0	0	0	0	0
Arya	0	0	0	1	1	0	1	0	0	0	0
Austen	0	0	0	0	1	0	0	0	0	0	0
Babu	0	0	1	1	1	0	1	0	0	0	0
Bajgai	0	0	0	0	1	0	0	0	0	0	0
Balaskas	0	0	0	0	0	1	0	0	0	0	0
Bapat	0	0	0	0	0	1	0	0	0	0	0
Batliwala	0	0	0	0	1	0	1	0	0	0	0
Becker	0	0	0	0	0	0	0	0	0	0	0
Bodker	0	0	0	0	1	0	0	1	0	0	0
Bouzas	0	0	0	0	1	0	0	0	1	0	0
Caccamise	1	0	0	0	1	1	0	0	0	0	0
Chaudhary	0	0	0	1	1	0	1	0	0	0	0
Chawla	1	0	0	0	0	1	0	0	0	0	0
Christiansen	1	0	0	0	1	0	0	0	0	0	0
Conrath	0	0	0	0	0	1	0	0	0	0	0
Das	0	0	0	1	0	0	1	0	0	0	0
Demir	1	0	0	0	1	0	0	0	0	0	0
Do Vale	0	0	0	0	1	0	0	0	0	0	0

Dowling	1	0	0	0	1	0	0	0	0	0	0
Elizalde	0	0	0	0	1	0	0	0	0	0	0
Escano	0	0	0	0	0	0	0	0	0	0	0
Font	0	0	0	0	1	0	1	1	0	1	0
Gaur	0	0	0	0	0	1	0	0	0	0	0
Genc	0	0	0	0	0	0	0	0	0	0	1
Gong	0	0	0	0	0	1	0	0	0	0	0
Guclu	0	0	0	0	0	1	0	0	0	0	0
Gupta	0	0	0	0	0	1	0	0	0	0	0
Harada	0	0	0	0	0	0	0	0	0	0	0
Hayasaka	1	0	0	0	0	0	0	0	0	0	0
Hayasaka	1	0	0	0	0	0	0	0	0	0	0
Herath	0	0	0	0	0	0	0	0	0	0	0
Ibrahim	1	0	0	0	0	1	0	0	0	0	0
Jain IS	1	0	0	1	1	0	0	0	0	0	0
Jain R	0	0	0	1	0	0	1	0	0	0	0
Jain VK	0	0	0	0	1	0	0	0	0	0	0
Kaliaperumal	0	0	0	1	1	0	1	0	0	0	0
Kancherla	0	0	0	0	1	0	0	0	0	0	0
Kannan	0	0	0	1	0	1	0	0	0	0	0
Kapran	0	0	0	0	1	0	0	0	0	0	0
Karam	0	0	0	0	0	0	0	0	0	0	0
Karam	1	0	0	0	0	0	0	0	0	0	0
Karam	0	0	0	1	1	0	0	1	0	0	0
Karam	1	0	0	0	0	0	0	0	0	0	0

Karam	1	0	0	0	0	0	0	0	0	0	0
Karam	0	0	1	0	1	0	0	0	0	0	0
Karam	0	0	0	0	1	0	1	1	0	0	0
Karwatowski	0	0	0	1	0	1	0	0	0	0	0
Kaushik	0	0	0	0	1	0	0	0	0	0	0
Kausman	0	0	0	0	0	0	0	0	0	0	0
Kavitha	0	0	0	1	1	0	1	0	0	0	0
Kim	1	0	0	1	0	1	0	0	0	0	0
Kimura	1	0	0	0	0	1	0	0	0	0	0
Kinoshita	0	0	0	0	0	0	0	0	0	0	0
Koz	0	0	0	0	1	0	0	0	0	0	0
Kumar	1	0	0	0	1	0	1	0	0	0	0
Kuwahara	0	0	0	0	1	0	0	0	0	0	0
Larrazabal	0	0	0	0	1	0	0	0	0	0	0
Lee GI	0	0	0	0	1	0	0	1	0	0	0
Lee JC	1	0	0	0	0	0	0	1	0	0	0
Leonard	1	0	0	0	0	0	0	0	0	0	0
Lewis	1	0	0	0	0	0	0	0	0	0	0
Lim	0	0	0	0	0	1	0	0	0	0	0
Mahajan	0	0	0	0	1	0	0	0	0	0	0
Mahajan	0	0	0	0	0	1	0	0	0	0	0
Mahajan	0	0	0	0	1	0	0	1	0	0	0
Mahajan	0	0	0	1	1	0	0	0	0	0	0
Mahajan	0	0	0	0	1	0	0	0	0	0	0
Mahendradas	0	0	0	0	0	0	0	0	0	0	0

Mashru	0	0	0	0	0	1	0	0	0	0	0
Matalia	0	0	0	0	0	1	0	0	0	0	0
Matsumoto-Otake	0	0	0	0	1	0	0	0	0	0	0
McDonald	0	0	0	0	1	0	0	1	0	0	0
Milea	0	0	0	0	1	0	0	0	0	0	0
Moncada	1	0	0	0	0	1	0	1	0	0	0
Natraj	0	0	1	0	1	1	0	0	0	0	0
Nithyanandam	1	0	0	0	0	1	0	0	0	0	0
Noel	0	0	0	0	1	0	0	0	0	0	0
Noel	1	0	0	0	0	0	0	0	0	0	0
Noel	0	0	0	0	1	0	0	0	0	0	0
Ostler	0	0	0	0	1	0	0	0	0	0	0
Padhy	0	0	0	0	1	0	0	0	0	0	0
Pahwa	0	0	0	0	1	0	0	0	0	0	0
Pallangyo	0	0	0	1	1	0	1	0	0	0	0
Paterson	0	0	0	0	1	0	0	0	0	0	0
Paul	1	0	0	0	0	0	0	0	0	0	0
Pelegrin	1	0	0	0	1	1	0	0	0	0	0
Peter	0	0	0	0	1	0	0	0	0	0	0
Peter	0	0	0	0	1	0	0	0	0	0	0
Peter	0	0	0	0	1	0	0	0	0	0	0
Peter	0	0	0	0	1	0	0	0	0	0	0
Peter	0	0	0	0	1	0	0	0	0	0	0

Rainer	0	0	0	0	0	0	0	0	0	0	0
Rahman	1	0	0	0	0	0	0	0	0	0	0
Rajshri	0	0	0	0	1	0	0	0	0	0	0
Ramteke	1	1	0	0	0	1	0	0	0	0	0
Reddy	0	0	0	0	1	0	0	0	0	0	0
Rodriguez	0	0	0	0	1	0	0	0	0	0	0
Sakthiswary	0	0	0	0	1	0	0	0	0	0	0
Santhanam	0	0	0	0	0	1	0	0	0	0	0
Setty	0	0	0	0	1	1	0	0	0	0	0
Shailaja	0	0	0	1	1	0	0	0	0	0	0
Shrestha	0	0	0	0	0	1	0	0	0	0	0
Shukla	0	0	0	1	0	0	1	0	0	0	0
Smith	1	0	0	0	0	0	1	0	0	0	0
Stone	0	0	0	1	1	0	0	0	0	0	0
Strauss	1	0	0	0	0	0	0	0	0	0	0
Subira	0	0	0	0	1	0	0	0	0	0	0
Suematsu	0	0	0	0	1	0	0	0	0	0	0
Suh	0	0	0	0	1	0	0	0	0	0	0
Sureja	0	0	0	1	0	0	1	0	0	0	0
Tani	0	0	0	0	0	0	0	0	0	0	0
Taylan	0	1	1	0	0	0	0	0	0	0	0
Tian	1	0	0	0	0	0	0	0	0	0	0
Topcuoglu	1	0	0	1	0	0	0	0	0	0	0
Torun	0	0	0	0	1	0	0	0	0	0	0
Ueno	0	0	0	0	0	0	0	0	0	0	0

Wu	0	0	0	0	0	1	0	0	0	0	0
Zahaf	0	0	0	0	0	1	0	0	0	0	0
Zeng	1	0	0	0	1	0	0	0	0	0	0

Abbreviations: VA – visual acuity

Table S2. Ocular manifestations of TA.

First author	Retinal ischemia	Hypertensive retinopathy	Scelritis	Keratitis	Atypival THS	RAO	Cataract	Uveitis	ERM	Episcleritis	Facial n. palsy	Optic neuro-pathy	Accuired ocular motor apraxia	RVO	Ptosis	Ophthalmo-plegia	Retinal vasculitis	Sterile corneal melt	MEWDS	Orbital pseudotumor	Exudative RD
Akhtar	0	0	1	0	0	0	0	0	0	0	0	0	0	0	0	0	0	0	0	0	0
Amer	0	0	1	0	0	0	0	0	0	0	0	0	0	0	0	0	0	0	0	0	0
Anguita	1	0	0	0	0	0	0	0	0	0	0	0	0	0	0	0	0	0	0	0	0
Arya	0	0	1	1	0	0	0	0	0	0	0	0	0	0	0	0	0	0	0	0	0
Austen	1	0	0	0	0	0	0	0	0	0	0	0	0	0	0	0	0	0	0	0	0
Babu	0	0	1	1	1	0	0	0	0	0	0	1	0	0	0	0	0	0	0	0	0
Bajgai	0	0	0	0	0	0	0	0	0	0	0	0	0	0	0	0	1	0	0	0	0
Balaskas	0	0	0	0	0	1	0	0	0	0	0	0	0	0	0	0	0	0	0	0	0
Bapat	0	0	0	0	0	0	1	0	0	0	0	0	0	0	0	0	0	0	0	0	0
Batliwala	1	0	0	0	0	0	0	0	0	0	0	0	0	0	0	0	0	0	0	0	0
Becker	0	0	0	0	0	0	0	1	0	0	0	0	0	0	0	0	0	0	0	0	0

Bodker	1	0	0	0	0	0	0	0	0	0	0	0	0	0	0	0	0	0	0	0	0
Bouzas	0	0	0	0	0	0	0	0	1	0	0	0	0	0	0	0	0	0	0	0	0
Caccamise	1	0	0	0	0	0	1	0	0	0	0	1	0	0	0	0	0	0	0	0	0
Chaudhary	0	0	1	0	0	0	1	0	0	0	0	0	0	0	0	0	0	0	0	0	0
Chawla	0	0	0	0	0	1	0	0	0	0	0	0	0	0	0	0	0	0	0	0	0
Christiansen	1	0	0	0	0	0	0	0	0	0	0	0	0	0	0	0	0	0	0	0	0
Conrath	0	0	0	0	0	0	0	0	0	0	0	0	0	1	0	0	0	0	0	0	0
Das	0	0	0	0	0	0	0	0	0	1	0	0	0	0	0	0	0	0	0	0	0
Demir	1	0	0	0	0	0	0	0	0	0	0	0	0	0	0	0	0	0	0	0	0
Do Vale	1	0	0	0	0	0	0	0	0	0	0	0	0	0	0	0	0	0	0	0	0
Dowling	1	0	0	0	0	0	0	0	0	0	0	0	0	0	0	0	0	0	0	0	0
Elizalde	1	0	0	0	0	0	0	0	0	0	0	0	0	0	0	0	0	0	0	0	0
Escano	1	0	0	0	0	0	0	0	0	0	0	0	0	0	0	0	0	0	0	0	0
Font	1	0	0	0	0	0	1	0	0	0	0	0	0	0	0	0	0	0	0	0	0
Gaur	1	0	0	0	0	0	0	0	0	0	1	1	0	0	0	0	0	0	0	0	0
Genc	0	0	0	0	0	0	0	0	0	0	0	0	1	0	1	0	0	0	0	0	0
Gong	1	0	0	0	0	0	0	0	0	0	0	0	0	0	0	0	0	0	0	0	0
Guclu	0	0	0	0	0	1	0	0	0	0	0	0	0	0	0	0	0	0	0	0	0
Gupta	0	0	0	0	0	1	0	0	0	0	0	0	0	1	0	0	0	0	0	0	0
Harada	0	0	0	0	0	0	1	0	0	0	0	0	0	0	0	0	0	0	0	0	0
Hayasaka	1	0	0	0	0	0	0	0	0	0	0	0	0	0	0	0	0	0	0	0	0
Hayasaka	1	0	0	0	0	0	0	0	0	0	0	0	0	0	0	0	0	0	0	0	0
Herath	0	0	0	0	0	0	0	0	0	0	0	0	0	0	1	1	0	0	0	0	0
Ibrahim	1	0	0	0	0	0	0	0	0	0	0	0	0	0	0	0	1	0	0	0	0

Jain IS	1	0	0	0	0	0	1	0	0	0	0	0	0	0	0	0	0	0	0	0	0
Jain R	0	0	1	0	0	0	0	0	0	0	0	0	0	0	0	0	0	0	0	0	0
Jain VK	1	0	0	0	0	0	1	0	0	0	0	0	0	0	0	0	0	0	0	0	0
Kaliaperumal	0	0	0	1	0	0	0	1	0	0	0	0	0	0	0	0	0	0	0	0	0
Kancherla	0	1	0	0	0	0	0	0	0	0	0	0	0	0	0	0	0	0	0	0	0
Kannan	1	0	0	0	0	1	0	0	0	0	0	0	0	0	0	0	0	0	0	0	0
Kapran	0	0	0	0	0	0	0	0	0	0	0	1	0	0	0	0	0	0	0	0	0
Karam	1	0	0	0	0	0	0	0	0	0	0	0	0	0	0	0	0	0	0	0	0
Karam	1	0	0	0	0	0	0	0	0	0	0	0	0	0	0	0	0	0	0	0	0
Karam	1	0	0	0	0	0	0	0	0	0	0	0	0	0	0	0	0	0	0	0	0
Karam	1	0	0	0	0	0	0	0	0	0	0	0	0	0	0	0	0	0	0	0	0
Karam	1	0	0	0	0	0	0	0	0	0	0	0	0	0	0	0	0	0	0	0	0
Karam	1	0	0	0	0	0	0	0	0	0	0	1	0	0	0	0	0	0	0	0	0
Karam	0	0	0	0	0	0	0	0	0	0	0	1	0	0	0	0	0	0	0	0	0
Karwatowski	1	0	0	0	0	0	0	0	0	0	0	0	0	0	0	0	0	0	0	0	0
Kaushik	0	0	0	0	0	1	0	0	0	0	0	0	0	0	0	0	0	0	0	0	0
Kausman	0	0	0	0	0	0	0	1	0	0	0	0	0	0	0	0	0	0	0	0	0
Kavitha	0	0	0	0	0	0	0	0	0	0	0	0	0	0	0	0	0	1	0	0	0
Kim	0	0	0	0	0	0	0	0	0	0	0	1	0	0	0	0	0	0	0	0	0
Kimura	1	0	0	0	0	0	0	0	0	0	0	1	0	0	0	0	0	0	0	0	0
Kinoshita	1	0	0	0	0	0	0	0	0	0	0	0	0	0	0	0	0	0	0	0	0
Koz	1	0	0	0	0	0	0	0	0	0	0	0	0	0	0	0	0	0	0	0	0
Kumar	1	0	0	0	0	0	1	0	0	0	0	0	0	0	0	0	0	0	0	0	0
Kuwahara	1	0	0	0	0	0	0	0	0	0	0	0	0	0	0	0	0	0	0	0	0

Larrazabal	1	0	0	0	0	0	1	0	0	0	1	1	0	0	0	0	0	0	0	0	0
Lee GI	1	0	0	0	0	0	0	0	0	0	0	0	0	0	0	0	0	0	0	0	0
Lee JC	1	0	0	0	0	0	0	0	0	0	0	0	0	0	0	0	0	0	0	0	0
Leonard	1	0	0	0	0	0	0	0	0	0	0	1	0	0	0	0	0	0	0	0	0
Lewis	1	0	0	0	0	0	0	0	0	0	0	0	0	0	0	0	0	0	0	0	0
Lim	0	0	0	0	0	1	0	0	0	0	0	0	0	0	0	0	0	0	0	0	0
Mahajan	1	0	0	0	0	0	0	0	0	0	0	1	0	0	0	0	0	0	0	0	0
Mahajan	1	0	0	0	0	0	0	0	0	0	0	0	0	0	0	0	0	0	0	0	0
Mahajan	1	0	0	0	0	0	1	0	0	0	0	0	0	0	0	0	0	0	0	0	0
Mahajan	1	0	0	0	0	0	0	0	0	0	0	0	0	0	0	0	0	0	0	0	0
Mahajan	1	0	0	0	0	0	1	0	0	0	0	0	0	0	0	0	0	0	0	0	0
Mahendradas	0	0	0	0	0	0	0	0	0	0	0	0	0	0	0	0	1	0	0	0	0
Mashru	0	0	0	0	0	1	0	0	0	0	0	0	0	0	0	0	0	0	0	0	0
Matalia	0	0	0	0	0	0	0	0	0	0	0	1	0	0	0	0	0	0	0	0	0
Matsumoto-Otake	1	0	0	0	0	0	0	0	0	0	0	0	0	0	0	0	0	0	0	0	0
McDonald	0	0	0	0	0	0	0	1	0	0	0	0	0	0	0	0	0	0	0	0	0
Milea	1	0	0	0	0	0	1	0	0	0	0	0	0	0	0	0	0	0	0	0	0
Moncada	1	0	0	0	0	0	0	0	0	0	0	0	0	0	0	0	0	0	0	0	0
Natraj	0	0	0	0	0	1	0	0	0	0	0	0	0	0	0	0	0	0	0	0	0
Nithyanandam	0	0	0	0	0	0	0	0	0	0	0	1	0	0	0	0	0	0	0	0	0
Noel	1	0	0	0	0	1	0	0	0	0	0	1	0	0	0	0	1	0	0	0	0
Noel	0	1	0	0	0	1	0	0	0	0	0	0	0	0	0	0	0	0	0	0	0
Noel	1	0	0	0	0	0	0	0	0	0	0	0	0	1	0	0	0	0	0	0	0
Ostler	1	0	0	0	0	0	0	0	0	0	0	0	0	0	0	0	0	0	0	0	0
Padhy	1	0	0	0	0	0	1	0	0	0	0	0	0	0	0	0	0	0	0	0	0

Pahwa	1	0	0	0	0	0	0	0	0	0	0	0	0	0	0	0	0	0	0	0	0
Pallangyo	1	0	0	0	0	0	0	0	0	0	0	0	0	0	0	0	0	0	0	0	0
Paterson	1	0	0	0	0	0	1	0	0	0	0	0	0	0	0	0	0	0	0	0	0
Paul	1	0	0	0	0	0	1	0	0	0	0	0	0	0	0	0	0	0	0	0	0
Pelegrin	1	0	0	0	0	0	1	0	0	0	0	1	0	0	0	0	0	0	0	0	0
Peter	1	0	0	0	0	0	0	0	0	0	0	1	0	0	0	0	0	0	0	0	0
Peter	1	0	0	0	0	0	0	0	0	0	0	0	0	0	0	0	0	0	0	0	0
Peter	0	1	0	0	0	0	1	0	0	0	0	1	0	0	0	0	0	0	0	0	0
Peter	0	0	0	0	0	0	0	0	0	0	0	0	0	0	0	0	0	0	0	0	1
Peter	0	1	0	0	0	0	0	0	0	0	0	0	0	0	0	0	0	0	0	0	0
Rainer	0	1	0	0	0	0	0	0	0	0	0	0	0	0	0	0	0	0	0	0	0
Rahman	1	0	0	0	0	0	0	0	0	0	0	0	0	0	0	0	0	0	0	0	0
Rajshri	1	0	0	0	0	1	0	0	0	0	0	1	0	0	0	0	0	0	0	0	0
Ramteke	0	0	0	0	0	1	0	0	0	0	0	0	0	0	0	0	0	0	0	0	0
Reddy	1	0	0	0	0	0	0	0	0	0	0	1	0	0	0	0	0	0	0	0	0
Rodriguez	0	1	0	0	0	0	0	0	0	0	0	0	0	0	0	0	0	0	0	0	0
Sakthiswary	1	0	0	0	0	0	0	0	0	0	0	0	0	0	0	0	0	0	0	0	0
Santhanam	1	0	0	0	0	0	0	0	0	0	0	1	0	0	0	0	0	0	0	0	0
Setty	0	0	0	0	0	1	0	0	0	0	0	0	0	0	0	0	0	0	0	0	0
Shailaja	1	0	0	0	0	0	0	0	0	0	0	0	0	0	0	0	0	0	0	0	0
Shrestha	0	0	0	0	0	1	0	0	0	0	0	0	0	0	0	0	0	0	0	0	0
Shukla	0	0	1	0	0	0	0	0	0	0	0	0	0	0	0	0	0	0	0	0	0
Smith	0	0	1	0	0	0	0	0	0	0	0	0	0	0	0	0	0	0	0	0	0
Stone	0	0	0	0	0	0	0	1	0	0	0	0	0	0	0	0	0	0	0	0	0

Strauss	1	0	0	0	0	0	1	0	0	0	0	0	0	0	0	0	0	0	0	0	0
Subira	1	0	0	0	0	0	0	0	0	0	0	0	0	0	0	0	0	0	0	0	0
Suematsu	0	0	0	0	0	0	0	0	0	0	0	0	0	0	0	0	0	0	1	0	0
Suh	0	0	0	0	0	0	0	0	0	0	0	0	0	1	0	0	0	0	0	0	0
Sureja	0	0	1	0	0	0	0	0	0	0	0	0	0	0	0	0	0	0	0	0	0
Tani	1	0	0	0	0	0	0	0	0	0	0	0	0	0	0	0	0	0	0	0	0
Taylan	0	0	0	0	0	0	0	0	0	0	0	0	0	0	0	0	0	0	0	1	0
Tian	0	0	0	0	0	0	0	0	0	0	0	1	0	0	0	0	0	0	0	0	0
Topcuoglu	1	0	0	0	0	0	0	0	0	0	0	0	0	0	0	0	0	0	0	0	0
Torun	0	1	0	0	0	0	0	0	0	0	0	0	0	0	0	0	0	0	0	0	0
Ueno	1	0	0	0	0	0	0	0	0	0	0	0	0	0	0	0	0	0	0	0	0
Wu	1	0	0	0	0	0	0	0	0	0	0	0	0	0	0	0	1	0	0	0	0
Zahaf	1	0	0	0	0	0	0	0	0	0	0	1	0	0	0	0	0	0	0	0	0
Zeng	1	0	0	0	0	0	0	0	0	0	0	0	0	0	0	0	0	0	0	0	0

Abbreviations: THS – Tolosa-Hunt syndrome; RAO – retinal artery occlusion; ERM – epiretinal membrane; n. – nerve; RVO – retinal vein occlusion; MEWDS – multiple evanescent white dot syndrome; RD – retinal detachment

Table S3. Systemic treatment in patients with ocular manifestations of TA.

First author	Systemic steroids	Vascular procedure	Mycophenolate mofetil	Azathioprine	CP	MTX	Leflunomide	ASA	mAb	Antiplatelet drugs	Anticoagulants	Antihypertensives
Akhtar	1	0	1	0	0	0	0	0	1	0	0	0
Amer	1	0	0	0	0	0	0	0	0	0	0	0
Anguita	1	0	1	0	0	0	0	0	0	0	0	0
Arya	1	0	0	0	0	0	0	0	0	0	0	0
Austen	0	1	0	0	0	0	0	0	0	0	0	0
Babu	1	1	0	1	0	0	0	0	0	0	0	0
Bajgai	1	1	0	1	0	0	0	0	0	0	0	0
Balaskas	1	0	0	0	0	0	0	0	0	0	0	0
Bapat	1	0	0	0	0	0	0	0	0	0	0	0
Batliwala	1	0	0	0	0	1	0	0	0	0	0	0
Becker	0	1	0	0	0	1	0	0	0	0	0	0
Bodker	0	0	0	0	0	0	0	0	0	0	0	0
Bouzas	1	0	0	0	0	0	0	0	0	0	0	0
Caccamise	0	0	0	0	0	0	0	0	0	0	0	0
Chaudhary	1	0	0	0	0	1	0	0	0	0	0	1
Chawla	1	0	0	0	0	0	0	1	0	0	0	0
Christiansen	1	1	0	0	0	1	0	0	1	1	0	0
Conrath	1	1	0	0	0	0	0	1	0	0	1	0
Das	1	0	0	0	0	1	0	0	0	0	0	0
Demir	1	0	0	0	0	1	0	0	0	0	0	0
Do Vale	0	0	0	0	0	0	0	0	0	0	0	0

Dowling	0	1	0	0	0	0	0	0	0	0	0	0
Elizalde	0	0	0	0	0	0	0	0	0	0	0	0
Escano	0	0	0	0	0	0	0	0	0	0	0	0
Font	0	0	0	0	0	0	0	0	0	0	0	0
Gaur	1	0	0	0	0	0	0	0	0	0	0	0
Genc	0	0	0	0	0	0	0	0	0	0	0	0
Gong	1	0	0	0	0	0	1	1	0	1	0	0
Guclu	1	0	0	0	0	1	0	0	0	0	0	0
Gupta	1	1	0	0	0	1	0	0	0	1	1	1
Harada	1	0	0	0	0	0	0	0	0	0	0	0
Hayasaka	1	0	0	0	0	0	0	0	0	0	0	0
Hayasaka	1	0	0	0	0	0	0	0	0	0	0	0
Herath	1	0	0	1	0	0	0	1	0	0	0	0
Ibrahim	1	1	0	0	1	1	0	0	1	0	0	0
Jain IS	0	0	0	0	0	0	0	0	0	0	0	0
Jain R	1	1	0	0	1	0	0	0	0	0	0	1
Jain VK	0	1	0	0	0	0	0	0	0	0	0	0
Kaliaperumal	1	0	0	0	0	0	0	0	0	0	0	1
Kancherla	1	0	0	0	0	1	0	1	0	1	0	1
Kannan	1	0	0	0	0	1	0	0	0	0	0	0
Kapran	1	1	0	0	0	0	0	0	0	0	0	0
Karam	0	0	0	0	0	0	0	0	0	0	0	0
Karam	1	1	0	0	0	0	0	1	0	0	0	0
Karam	1	0	0	0	0	0	0	1	0	0	0	0
Karam	1	0	0	0	0	0	0	0	0	0	0	0

Karam	1	0	0	1	0	0	0	0	0	0	0	0
Karam	1	0	0	0	0	0	0	0	0	0	0	0
Karam	1	0	0	0	0	0	0	0	0	0	0	0
Karwatowski	0	0	0	0	0	0	0	0	0	0	0	0
Kaushik	1	0	0	0	0	0	0	0	0	0	0	0
Kausman	1	1	0	0	1	1	0	1	0	0	0	1
Kavitha	1	0	0	0	0	0	0	0	0	0	0	0
Kim	1	0	0	0	0	0	0	0	0	0	0	0
Kimura	1	1	0	0	0	0	0	0	0	0	0	0
Kinoshita	0	1	0	0	0	0	0	0	0	0	0	0
Koz	1	0	0	0	0	1	0	0	0	0	0	0
Kumar	0	0	0	0	0	0	0	0	0	0	0	0
Kuwahara	1	0	0	0	0	0	0	0	0	0	0	0
Larrazabal	1	0	0	0	0	0	0	1	0	0	0	0
Lee GI	1	0	0	0	0	0	0	0	0	0	0	0
Lee JC	0	1	0	0	0	0	0	0	0	0	0	0
Leonard	0	1	0	0	0	0	0	0	0	0	0	0
Lewis	1	1	0	0	0	0	0	1	0	0	1	0
Lim	1	0	0	0	0	1	0	0	0	0	0	0
Mahajan	1	0	0	0	0	1	0	0	0	0	0	0
Mahajan	0	0	0	0	0	0	0	0	0	0	0	0
Mahajan	1	0	0	0	0	1	0	0	0	0	0	0
Mahajan	0	0	0	0	0	0	0	0	0	0	0	0
Mahajan	0	0	0	0	0	0	0	0	0	0	0	0
Mahendradas	0	0	0	0	0	0	0	0	0	0	0	0

Mashru	1	0	0	0	0	1	0	0	0	0	0	0
Matalia	0	1	0	0	0	0	0	0	0	0	0	0
Matsumoto-Otake	1	0	0	0	0	0	0	0	0	1	0	0
McDonald	1	1	0	0	1	1	0	1	0	0	0	0
Milea	0	1	0	0	0	0	0	0	0	0	0	0
Moncada	1	1	0	0	0	0	0	1	0	0	0	1
Natraj	1	1	0	0	0	1	0	0	0	0	0	0
Nithyanandam	1	0	0	0	0	0	0	0	0	0	0	0
Noel	1	0	0	0	0	1	0	0	1	0	1	0
Noel	1	0	1	0	0	0	0	0	0	0	0	0
Noel	1	0	0	0	0	1	0	1	0	0	0	0
Ostler	0	0	0	0	0	0	0	0	0	0	0	0
Padhy	0	0	0	0	0	0	0	0	0	0	0	0
Pahwa	1	0	0	0	0	0	0	0	0	0	0	0
Pallangyo	1	0	0	0	0	1	0	1	0	0	0	0
Paterson	0	0	0	0	0	0	0	0	0	0	1	0
Paul	0	0	0	0	0	0	0	0	0	0	0	0
Pelegrin	1	0	0	0	0	1	0	1	0	0	0	0
Peter	1	1	1	0	0	0	0	1	0	1	0	0
Peter	0	0	0	0	0	0	0	0	0	0	0	0
Peter	0	1	0	0	0	0	0	0	0	0	0	0
Peter	0	1	0	0	0	0	0	0	0	0	0	1
Peter	0	1	0	0	0	0	0	0	0	0	0	1
Rainer	1	1	0	0	0	0	0	0	1	0	0	0

Rahman	1	0	1	1	1	1	0	0	0	0	0	0
Rajshri	1	0	0	0	0	0	0	0	0	0	0	0
Ramteke	1	0	0	0	0	0	0	1	0	1	1	0
Reddy	0	1	0	0	0	0	0	0	0	0	1	0
Rodriguez	1	0	0	0	0	0	0	0	0	0	0	1
Sakthiswary	1	0	0	0	0	1	0	0	0	0	1	0
Santhanam	1	0	0	0	0	1	0	0	0	1	0	1
Setty	1	1	0	0	0	1	0	0	0	0	0	0
Shailaja	1	0	0	0	0	0	0	0	0	0	0	0
Shrestha	1	0	1	0	0	0	0	1	0	0	0	0
Shukla	1	1	0	0	0	1	0	0	0	0	0	0
Smith	1	1	0	0	0	1	0	1	0	0	0	0
Stone	1	1	0	0	0	1	0	0	0	0	0	0
Strauss	0	0	0	0	0	0	0	0	0	0	0	0
Subira	0	1	0	0	0	0	0	0	0	0	0	0
Suematsu	1	0	0	0	0	1	0	0	1	0	0	0
Suh	0	0	0	0	0	0	0	0	0	0	0	0
Sureja	1	0	0	0	0	1	0	0	0	0	0	0
Tani	0	0	0	0	0	0	0	0	0	0	0	0
Taylan	1	0	0	0	1	0	0	0	0	0	0	0
Tian	1	1	0	0	0	0	0	0	0	0	0	0
Topcuoglu	1	0	0	0	0	0	0	0	0	0	0	0
Torun	1	1	0	1	0	1	0	1	1	0	0	1
Ueno	0	1	0	0	0	0	0	0	0	0	0	0
Wu	1	0	0	1	0	0	0	1	1	1	1	0

Zahaf	1	0	0	1	0	0	0	0	1	0	0	0
Zeng	1	1	0	0	1	0	0	1	0	0	0	0

Abbreviations: CP – cyclophosphamide; MTX – methotrexate; ASA – acetylsalicylic acid; mAb – monoclonal antibody
